# Overlap Between the General Factor of Personality and Trait Emotional Intelligence: A Genetic Correlation Study

**DOI:** 10.1007/s10519-017-9885-8

**Published:** 2017-12-20

**Authors:** Dimitri van der Linden, Julie A. Schermer, Eveline de Zeeuw, Curtis S. Dunkel, Keri A. Pekaar, Arnold B. Bakker, Philip A. Vernon, K. V. Petrides

**Affiliations:** 1 Department of Psychology, Education, and Child Studies, Erasmus University Rotterdam, P.O. Box 9104, 3000 DR Rotterdam, The Netherlands; 2 Management and Organizational Studies, University of Western Ontario, London, Canada; 3 Department of Biological Psychology, Free University Amsterdam, Amsterdam, The Netherlands; 4 Department of Psychology, Western Illinois University, Macomb, USA; 5 Department of Psychology, University of Western Ontario, London, Canada; 6 London Psychometric Laboratory, University College London, London, UK

**Keywords:** General factor of personality, Trait emotional intelligence, Heritability, Twins, Genetic correlation, TEIQue

## Abstract

A previous meta-analysis (Van der Linden et al., Psychol Bull 143:36–52, 2017) showed that the General Factor of Personality (GFP) overlaps with ability as well as trait emotional intelligence (EI). The correlation between trait EI and the GFP was so high (*ρ* = *0*.88) in that meta-analysis that these two may be considered virtually identical constructs. The present study builds on these findings by examining whether the strong phenotypic correlation between the GFP and trait EI has a genetic component. In a sample of monozygotic and dizygotic twins, the heritability estimates for the GFP and trait EI were 53 and 45%, respectively. Moreover, there was a strong genetic correlation of *r* = .90 between the GFP and trait EI. Additional analyses suggested that a substantial proportion of the genetic correlations reflects non-additive genetic effects (e.g., dominance and epistasis). These findings are discussed in light of evolutionary accounts of the GFP.

## Introduction

The scientific search for the basic personality dimensions has led to several models consisting of various, presumably independent, factors. One of the most prominent models in this area is the Big Five model (Goldberg 1981), which assumes that personality can best be described by five basic factors, namely openness to experience, conscientiousness, extraversion, agreeableness, and neuroticism, or its reverse, emotional stability. Using this five factor model, there is a growing body of literature showing that personality traits are not independent from each other; rather, they are associated such that positively valenced traits typically display positive intercorrelations (e.g., Figueredo et al. 2004; Musek 2007; Rushton and Irwing 2011; Van der Linden et al. 2010a, b). Thus, personality traits seem to share a relevant proportion of their variance, which has been labelled as the General Factor of Personality or GFP (Figueredo et al. 2004; Rushton et al. 2008). In terms of the Big Five model, high-GFP individuals would be, on average, open-minded, diligent, sociable, friendly, and emotionally stable.

The GFP has now been recovered in not only the Big Five, but also various other personality models (e.g., Figueredo et al. 2004; Loehlin 2012; Musek 2007; Rushton and Irwing 2011; Van der Linden et al. 2010a, b), yet questions about its nature remain. Several scholars posit that the GFP is substantive and reflects a broad trait correlated with important life domains, such as occupational behavior, mental health, and social behavior (Dunkel and Van der Linden 2014; Loehlin 2012; Van der Linden et al. 2010a, b). Others tend to favor the interpretation of the GFP as mainly statistical or systematic bias caused by the methods personality is measured (e.g., Bäckström et al. 2009). For example, due to a tendency towards socially desirable responding or an inflated self-image, some individuals may acquire more socially desirable personality profiles (leading to higher GFP scores) even though this does not necessarily reflect how they genuinely behave (Riemann and Kandler 2010). Thus, in this latter view, the GFP could be considered an artifact that interferes with the optimal assessment of an individual’s “true personality”.

In a review of the literature, Van der Linden et al. (2016) extensively discussed the arguments for the substantive as well as the artifact explanations of the GFP and concluded that, although some level of artifact or bias might be expected, the GFP most likely reflects a substantive factor (see also Dunkel et al. 2016). One of the main arguments for this conclusion was that the GFP is associated with a wide range of objective or other-rated outcome measures of social effectiveness. That is, the GFP has been positively related to popularity and likability among student classmates (Van der Linden et al. 2010b), social behavior during negotiations (Dunkel et al. 2014a, b), and job performance as rated by supervisors (Van der Linden et al. 2010a). GFP scores correlate negatively with the odds of experiencing mental disorders (Rushton and Irwing 2011) and engaging in delinquent behavior (Van der Linden et al. 2015a, b). The leading substantive interpretation of the GFP is that it mainly reflects general social effectiveness (Dunkel and Van der Linden 2014; Loehlin 2012). High-GFP individuals are characterized by knowledge of socially desirable behaviors and the ability to act accordingly, thereby increasing their probability of achieving their personal goals (e.g., getting a desired job or finding a partner).

In line with this, it has been argued and shown that the GFP overlaps with a well-established construct in psychology, viz., emotional intelligence (EI; Veselka et al. 2009; Van der Linden et al. 2012). Similar to the GFP, a core aspect of EI involves knowing how to act in order to optimize the likelihood of attaining social or personal goals (Matthews et al. 2004). Thus, an important aspect of EI relates to the ability to deal effectively with social demands. Strong support for the link between the GFP and EI has been provided by a recent meta-analysis that took into account ability as well as trait EI measures (Van der Linden et al. 2017). In that study, the estimated true correlation between the GFP and ability EI (as measured with maximum ability tests) was approximately 0.28. The estimated true correlation with trait EI was much higher, at 0.88. Trait EI is formally defined as a constellation of emotional perceptions assessed via questionnaires and rating scales (Petrides et al. 2007). Subsequently, it was concluded that the GFP and trait EI are very similar and possibly identical constructs.

The fact that trait and ability EI are differentially related to the GFP is unsurprising because extensive validity differences between the two constructs was one of the key reasons why they were conceptually separated in the first place (Petrides 2011; Petrides et al. 2004). Ability EI researchers categorize EI as part of the cognitive ability domain, whereas trait EI researchers emphasize the overlap between EI and personality traits. Meta-analytic research has revealed that trait EI measures tend to perform much better than ability EI measures in predicting important life outcomes (Martins et al. 2010; O’Boyle et al. 2011).

The discovery of the large variance overlap between trait EI and the GFP constituted an important breakthrough in establishing the nature of the former construct’s relationship to personality (Van der Linden et al. 2017). Nevertheless, a relevant question that has not yet been addressed concerns the extent to which the GFP-trait EI overlap is genetic. Addressing this question would be a logical next step in the broader effort to gain more insight into the etiology of the GFP.

### The Genetics of the GFP and Trait EI

Previous research has established the substantial genetic component of the GFP. Figueredo et al. (2004), Rushton et al. (2008), and Veselka et al. (2009) were among the first to estimate the heritability of the GFP at approximately 50% (see also Bell et al. 2012; Loehlin 2012). This value resembles the heritability estimates reported for the Big Five (e.g., Bouchard 1993; Jang et al. 1996; Loehlin 2012).

Figueredo and Rushton (2009) evaluated the behavioral genetic evidence and concluded that the GFP may have been shaped by evolutionary selective processes. Their conclusion was based on the finding that a considerable proportion (50%) of the genetic variance in the GFP was non-additive. Additive genetic variance implies that the phenotype of a specific trait can be directly inferred by summing up the effects of multiple genes. Non-additive genetic variance, on the other hand, involves more complex interactions between genes in determining the phenotype (Falconer 1989). In behavioral genetic research, it is posited that traits under recent natural selection have a higher ratio of non-additive to additive genetic variation (e.g., Figueredo and Rushton 2009) because selection processes delete additive genetic variation faster than non-additive genetic variation (Fisher 1954).

Some scholars have cautioned against associating evolutionary significance with non-additive genetic variance because this variance is often underestimated in twin studies (Keller et al. 2010). Moreover, non-additive genetic variance does not reveal the exact *type* of selection that has taken place, such as balancing selection (the trait is maintained in the population in an optimal proportion) or mutation drift (the presence of DNA mutations varies among individuals according to chance).

Nevertheless, a range of genetic studies have supported the idea of selective influences on the GFP and have provided clues about what type of selection may have occurred, viz., directional selection whereby selective pressures are associated with a clear advantage for individuals scoring higher on the trait (e.g., Figueredo and Rushton 2009; Figueredo et al. 2015). Directional selection certainly makes sense when the GFP is considered as a social effectiveness factor. Individuals who, during human history, knew how to deal with others would have benefited from this advantage, leading to higher survivability, and ultimately, more offspring (Figueredo et al. 2015).

Verweij et al. (2012) found that GFP scores were negatively related to inbreeding, which is evidence for directional selection. Inbreeding refers to offspring from individuals from a common ancestry (e.g., cousins). The presence of inbreeding effects indicates directional selection because inbreeding causes an accumulation of negative mutations in a population (i.e., the mutation load). Verweij et al. (2012) analyzed the genotypes of 5530 individuals on so-called runs of homozygosity, which estimate the degree of genetic material inherited from common ancestors. They found that those with more runs of homozygosity had lower GFP scores.

The heritability of trait EI has also been examined in previous studies and estimated to be around 40% (Vernon et al. 2008). The fact that trait EI and the GFP show a very high phenotypic correlation and that both constructs have heritable components suggests that some part of their overlap may be genetic. Nevertheless, this information does not necessary imply a strong genetic correlation. In theory, two correlated traits can both have high heritabilities, but their overlap may be non-genetic (e.g., Eaves et al. 1996). Yet, based on the extremely large phenotypic overlap found in a previous meta-analysis (Van der Linden et al. 2017) and the hypothesis that trait EI and the GFP may share common psychological mechanisms (e.g., social knowledge and skills), it is conceivable that their correlation does have a substantial genetic component. For example, if a set of genes influenced a broad psychological mechanism jointly involved in the manifestation of personality and trait EI, then we may speak of *pleiotropy* (e.g., Keller et al. 2010); genes that affect multiple phenotypic traits. In such a case, the GFP and trait EI would be expected to show a positive genetic correlation.

Vernon et al. (2008) reported substantial genetic correlations between global trait EI and many of its factors and facets on the one hand, and the Big Five personality dimensions on the other. Nevertheless, to our knowledge, no previous studies have directly tested the genetic correlation between the GFP and trait EI. Accordingly, we examined it on data from a sample of adult twins in Canada and the United States (Vernon et al. 2008).

## Method

### Participants

Participants were 316 adult twin pairs (mean age = 40.25 years, SD = 17.1 years; range 15–92 years), recruited primarily via newspaper advertisements across Canada and the United States. Of these, 44 pairs were monozygotic (MZ) male twins, 192 pairs were MZ female twins, nine pairs were dizygotic (DZ) male twins, and 71 pairs were DZ female twins. There were no age differences between the four zygosity groups (*F*(3,309) = 1.23, *p* = .298).

### Measures and Procedure

#### Personality

Twins completed the NEO-PI-R (Costa and McCrae 1992), a widely used instrument to measure the Five-Factor model personality dimensions (openness to experience, conscientiousness, extraversion, agreeableness, and neuroticism). The questionnaire consists of 240 items and has excellent psychometric properties. In the present sample, reliabilities (Cronbach’s Alphas) were, 0.74, 0.78, 0.76, 0.76, and 0.86 for O, C, E, A, and N, respectively. The Big Five were used to derive a general factor of personality as described below.

#### Trait EI

Twins also completed the TEIQue (Petrides 2009), which consists of 153 items predicated on trait EI theory and covering the sampling domain of the construct comprehensively. The TEIQue yields scores on 15 facets, four factors, and global trait EI. In line with the meta-analysis of phenotypic correlations (Van der Linden et al. 2017) only the global trait EI score was examined in the present study (for detailed information about the trait EI factors and facets, see Vernon et al. 2008). The sample reliability of global trait EI was 0.90, calculated from the 15 facets.

### Genetic Analyses

**Fig. 1:**
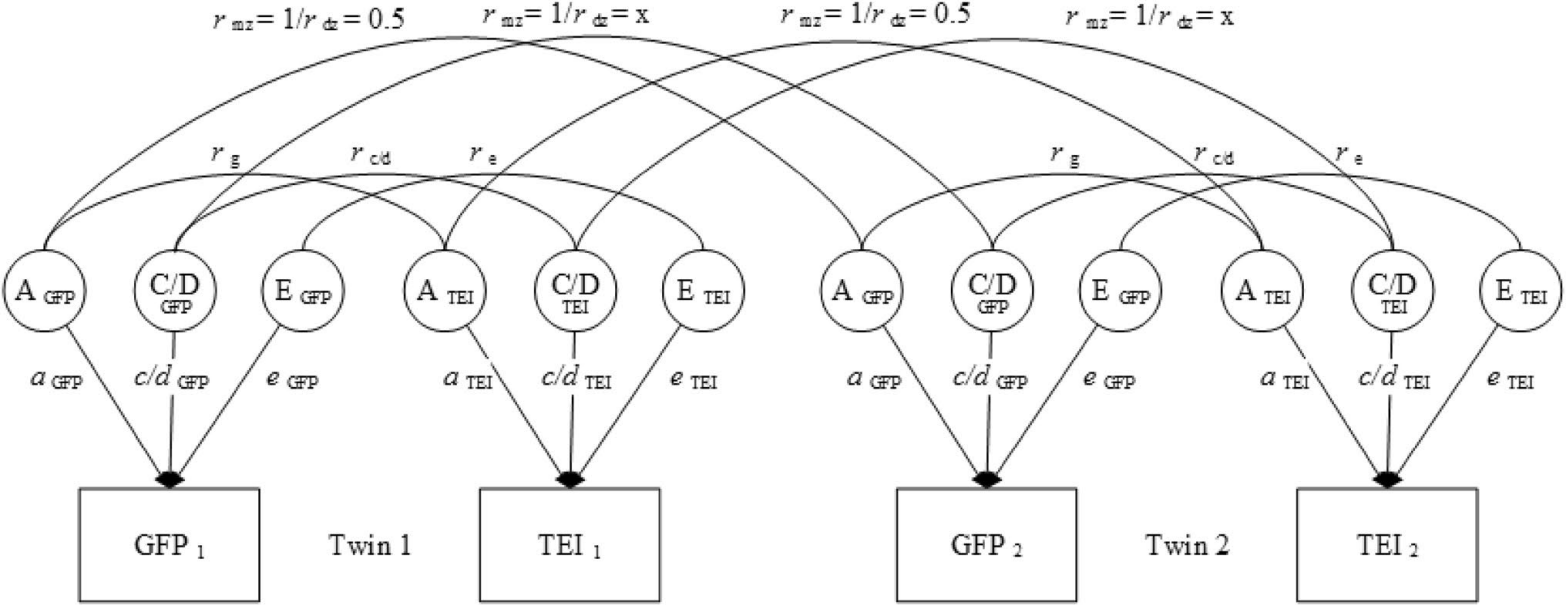
Illustration of ACE and ADE models tested. *GFP* general factor of personality, *TEI* trait emotional intelligence. The value of x in figure depends on the model tested. In the ACE model x = 1, in the ADE model x = 0.25. C/D indicates C in the ACE model and D in the ADE model

Twin studies, which capitalize on the difference in genetic relatedness between MZ and DZ twins, can be used to explore the underlying etiology of the overlap between the GFP and trait EI. First, a saturated model that freely estimated all parameters, i.e. means, variances and covariances, separately for the two zygosity groups (MZ and DZ) was fitted to the data using maximum likelihood raw data estimation in the R package OpenMx version 2.7.9 (Neale et al. 2016). Phenotypic correlations, within-trait and across-trait twin correlations were calculated by standardizing this model. Next, bivariate genetic models (ACE and ADE, see Fig. 1) were fitted to the data. In these models, the variance in the GFP and trait EI along with their covariance were decomposed into (co)variance due to additive genetic effects (A), to dominant genetic effects or to common environmental effects (C), and unique environmental effects (E). The variance components are expected to correlate differently for MZ and DZ twins, as they differ in their genetic resemblance. MZ twins share nearly all their genes while DZ twins are genetically just as alike as regular siblings (~ 50%). For an extended description of the twin method and its assumptions, see Posthuma et al. (2003).

Subsequently, the model that best represented the data (ACE or ADE model) was determined using the Akaike criterion, as these models are not nested. Finally, the significance of the different variance components was tested by dropping them from the model. For example, in the DE model, the path loadings of the additive genetic effects on GFP and trait EI (i.e., a1, a21, a2) were fixed to 0. The difference in model fit between the nested models was assessed with a log-likelihood ratio test (LRT). Constraints were kept when a more restrictive model did not significantly decrease the goodness of fit. The best fitting model was used to compute the genetic and environmental correlation between the two constructs, which estimate the extent to which their phenotypic (observed) correlations are attributable to the same genes and/or the same environmental factors.

## Results

In accordance with most previous studies (e.g., Dunkel et al. 2014a, b; Rushton and Irwing 2011; Van der Linden et al. 2010a, b; Veselka et al. 2009), the GFP was extracted using Principal Axis Factoring of the NEO-PI-R-based five factor scores. To prevent violations of independence, individual twins within a pair were examined separately as well as together. Table 1 contains the GFP loadings for the five personality factors per twin (individuals were randomly assigned as either “Twin 1 or Twin 2”) as well as for the combined sample. The GFP in this sample was mainly characterized by emotional stability (low neuroticism), conscientiousness, agreeableness, and extraversion. The loading on openness was relatively low. This is in line with a meta-analysis showing that openness has the least stable relationship with the GFP, with loadings fluctuating across samples and instrument characteristics (Van der Linden et al. 2010a, b). The fluctuations may also be related to the fact that openness (or its closely related concept of intellect) is the least clear and stable trait in the Big Five model (van der Linden et al. 2010a, b). A GFP score was computed for each individual using a weighted (by factor loading) linear aggregate. In order to test the consistency of the GFP in relation to sex, we conducted parallel analyses in males and females. These showed that the GFP had highly similar characteristics in both sexes with respect to the level of variance explained (33 and 28% for males and females, respectively) and the individual contribution (factor loadings) of the Big Five dimensions (0.08, 0.53, 0.35, 0.42, − 0.89 in males and 0.02, 0.72, 0.37, 0.51, − 0.88, in females) for O, C, E, A, and N, respectively.

**Table 1:** GFP extraction details (factor loadings)

	Twin 1	Twin 2	Total sample
Neuroticism	− 0.83	− 0.77	− 0.80
Extraversion	0.32	0.41	0.36
Openness	0.01	0.17	0.08
Agreeableness	0.40	0.46	0.43
Conscientiousness	0.60	0.59	0.60
Variance explained by the GFP (%)	26.59	26.92	26.57

The phenotypic correlation between the GFP and the global trait EI score was 0.71, which replicates findings in Van der Linden et al. (2017). For the GFP, the within-trait correlation was 0.51 and 0.04 for MZ and DZ twins, respectively, while for trait EI, the corresponding values were 0.43 and 0.03. These results suggest a relatively strong influence of non-additive genetic effects on both traits. In the present sample, the cross-trait cross-twin correlation (between one twin’s GFP score and their co-twin’s trait EI score) was 0.42 for the MZ pairs, but only − 0.04 for the DZ pairs, strongly suggesting the influence of genetic factors on the overlap between the two traits.

Even though the number of male and female twin pairs did not allow for direct and meaningful statistical comparisons between the sexes, the absolute values of the GFP-trait EI correlations for MZ and DZ twins were similar in both groups (0.53 and − 0.02 for males, and 0.45 and − 0.16 for females). This finding, in combination with the sex-similarity of the GFP characteristics reported above, and the fact that basic heritability estimates of personality (Vink et al. 2012) and trait EI (Veselka et al. 2009) do not differ between males and females, confirmed that it was possible to conduct the analyses on the whole sample.

The fairly low DZ twin correlations in this sample necessitated several decisions in the model fitting. Specifically, guidelines state that when the MZ correlation is more than twice the strength of the DZ correlation, this suggests the presence of non-additive genetic variance and hence an ADE model is recommended (Eaves et al. 1996). In the standard ADE model, the correlation between the dominant genetic effects is set to 0.25 for DZ twins and 1 for MZ twins. Yet, because the DZ twin correlations were close to zero this may be interpreted as evidence for epistasis. Epistasis refers to complex interactions between genes at different loci, for example, in which the phenotypical expression of one gene can mask the phenotypical expression of another (e.g., Cordell 2002; Phillips 2008). When many genes are involved in a trait, epistasis can considerably lower the DZ twin correlations. In line with this, one of the options was to apply an ADE model in which the correlation between the D factors is set to zero for DZ twins. Given the near-zero DZ correlations for both traits, it can be expected that such a model would provide an even better fit than the standard ADE model (because it is obviously more in line with the observed data). On the other hand, the literature, including several meta-analyses, shows that in complex traits, full epistasis is rather unlikely to explain all individual differences (Polderman et al. 2015; Risch 2000; Risch et al. 2009). Therefore, we restricted our main analyses to the more commonly used ADE model in which we assume DZ twins to share 25% of the D effects, which provides more conservative and general indications of the level of non-additive genetic variance (either dominance or epistasis).

The model fitting results (see Table 2) showed that the ADE model (*ep* = 22, *df* = 957, *− 2LL* = 1671.5, *AIC* = − 242.5) indeed was a better fit for the data than the ACE model (*ep* = 22, *df* = 957, *− 2LL* = 1676.2, *AIC* = − 237.8). This suggests that non-additive genetic effects had an influence on the GFP, trait EI and their overlap. The model fits also reveal that the influence of the common environment was negligible, which is in line with many other genetic studies on complex traits (Polderman et al. 2015).

**Table 2:** Model fitting statistics

	*ep*	− 2*ll*	*AIC*	*df*	compared to model	Δχ^2^	Δ*df*	*p*
0. Saturated	22	1646.2	− 245.8	946				
1. ADE	11	1671.5	− 242.5	957	0	25.3	11	0.008
2. ACE	11	1676.2	− 237.8	957	0	30.1	11	0.002
3. DE^a^	8	1671.9	− 248.1	963	1	0.4	3	0.940
4. E	5	1735.6	− 190.4	963	3	63.7	3	< 0.001

*ep* estimated parameters

^a^Best fitting model

Although the focus of the present study was on the genetic correlation between the GFP and trait EI, we consider it useful to report the basic heritability estimates of the two constructs (see also Bell et al. 2012; Vernon et al. 2008). The genetic component of the GFP was 53% (A = 3% and D = 50%). Forty-seven percent of the variance in the GFP could be attributed to non-shared environmental (E) factors and measurement error. For trait EI, 45% (A = 2% and D = 43%) of the variance was accounted for by genetic effects, while 55% by unique environmental effects.

In the present dataset, the effect of additive genetic variation (A) could be dropped from the ADE model. Thus, in the best fitting model (DE), in which the three parameters of A were fixed to zero, the genetic correlation was estimated at *r* = .90 (95% CI − 0.78 to 1.00) while the unique environmental correlation was *r* = .54 (95% CI 0.40–0.66) suggesting that all genetic covariance in the GFP-trait EI association could be attributed to non-additive genetic variance.

## Discussion

Building on a previous meta-analysis showing that the GFP and trait EI have a high phenotypic overlap of *ρ* = 0.88 (Van der Linden et al. 2017), the present study tested the extent to which this overlap has a common genetic factor. The findings confirm the presence of such a factor, as there was a strong genetic correlation of 0.90. It could be concluded that the GFP-trait EI overlap is mainly due to common genetic factors and non-shared environmental factors (which includes measurement error). The influence of shared environmental factors (e.g., parenting style and SES) was negligible.

In applying the ADE model, it was found that the additive effect (A) could be dropped, suggesting that the GFP-trait EI overlap mainly reflects non-additive genetic (D) and non-shared environmental (E) effects. It has been noted, however, that such a finding has to be interpreted with caution because it is considered unlikely that all genes for a specific behavior will be non-additive with no additive effects (Risch et al. 2009). It has been suggested that findings in which A can be dropped from the ADE model may be due to relatively low power (Neale and Cardon 2013; Risch 2000). In the present study, the sample size was indeed relatively small. On the other hand, it was also clear that in the ADE model, the additive genetic component (A) was very small, which makes power issues an unlikely explanation for the findings regarding this model.

Another possibility that has been proposed (Neale and Cardon 2013) is that variance of the main effects (A) may be aliased into the interaction effects (D). This implies that even though dropping the A is statistically valid, it may not fully reflect the true (causal) situation. Nevertheless, despite some points of debate regarding the relative contributions of A and D, at a minimum, it seems safe to conclude that, in the present sample, the genetic component in the GFP-EI overlap is substantial and likely holds an appreciable share of non-additive genetic variance. As also mentioned in the “Results” section, the relatively large difference between the correlations in the MZ and DZ twins suggests the presence of epistatic effects. Epistasis implies complex interactions between genes in the phenotypic expression (or non-expression) of traits. Given the breadth and complexity of social effectiveness and trait emotional intelligence, it is reasonable to hypothesize that they are not simple expressions of recessive and dominant genes, but regulated by many different genes that interact in various ways. Similar to our reasoning above, it is relevant to note that even though epistasis in complex traits is probable, it is unlikely that it can account for a lack of DZ twin correlations (e.g., Polderman et al. 2015; Risch et al. 2009). Therefore, given the relatively modest sample size, the safest conclusion to draw is that there are clear indications for the presence of dominance as well as epistasis effects.

All in all, the presence of non-additive genetic effects suggests that the GFP and trait EI may have been under recent natural selection (Falconer 1989, pp. 330–331; Figueredo and Rushton 2009; Fisher 1954). Specifically, during human history, high-GFP individuals may have been evaluated more favorably by others and may have more often been chosen as a partner, co-worker, or leader (Figueredo et al. 2004, 2007; Figueredo and Rushton 2009; Rushton et al. 2008). Such selection biases would have contributed to the reproductive fitness of these individuals, thus allowing evolutionary selective mechanisms to act on the GFP and linking personality to trait EI. Several evolutionary theories acknowledge that people with the ability to cooperate in groups (e.g., those with high trait EI) have indeed been more successful in competing over scarce resources (Geary 2005).

Even though the findings are in line with the hypothesis of natural selection for the GFP and trait EI, alternative explanations are possible. One such is that the results merely indicate the heritability of one very specific type of behavior, viz., the tendency to fake good on questionnaire items. If this tendency is heritable, it will apply equally to the GFP and trait EI, as both are operationalized mainly through questionnaires. In theory, this could lead to a genetic correlation between the GFP and trait EI even though both constructs would be artifactual, rather than substantive (for a discussion on response bias versus substance, see, for example, Bäckström et al. 2009; Irwing 2013; Van der Linden et al. 2016, 2017). However, it should be remembered that socially desirable responding does not solely reflect how participants answer questionnaire items, but a more general and genuine tendency to behave in socially desirable ways and to seek social approval (e.g., Connelly and Chang 2016). Darwin (1871) referred to this as “approval of one’s fellows” and considered it a generalized tendency in line with evolutionary selective pressure for cooperative and mutualistic behavior (for a discussion, see Figueredo et al. 2015). Consequently, it should be clear that even the social desirability account is consistent with our conceptualization of the GFP and trait EI as constructs relating to social effectiveness.

Future studies, with larger twin samples, may directly want to test for possible sex differences in the genetic GFP-trait EI correlation, although current evidence seems to indicate that there are none. In addition, further research on larger sample sizes is necessary in order to disentangle the alternative explanations for the genetic GFP-trait EI correlation in terms of substantive and response bias effects, and to establish their relative contribution.

The present study provides initial data on the genetic correlation between the GFP and trait EI, and further insight into their strong phenotypic correlation. The results also add to the growing body of literature on the evolutionary background of the GFP (Dunkel et al. 2014a, b; Figueredo et al. 2004, 2006, 2007; Rushton et al. 2008; Van der Linden et al. 2012, 2015a). With regard to trait EI, the literature has largely overlooked evolutionary accounts (although see Figueredo et al. 2011; Van der Linden et al. 2015a, b). As such, we are optimistic that the results of the present paper will inspire future research on the genetic and evolutionary background and development of this important individual-differences dimension.
